# Design and Performance Assessment of Biocompatible Capacitive Pressure Sensors with Circular and Square Geometries Using ANSYS Workbench

**DOI:** 10.3390/s25082423

**Published:** 2025-04-11

**Authors:** Md Shams Tabraiz Alam, Shabana Urooj, Abdul Quaiyum Ansari, Areiba Arif

**Affiliations:** 1 Department of Electrical Engineering, Jamia Millia Islamia, New Delhi 110025, India; shams.tabraiz2k6@gmail.com (M.S.T.A.); aqansari@jmi.ac.in (A.Q.A.); 2 Department of Electrical Engineering, College of Engineering, Princess Nourah Bint Abdulrahman University, P.O. Box 84428, Riyadh 11671, Saudi Arabia; 3 Jindal Global Business School, OP Jindal Global University, Sonipat 131001, India; phd18areiba@iima.ac.in

**Keywords:** capacitive pressure sensor, in-stent restenosis, biocompatible materials, sensor design, polydimethylsiloxane-PDMS

## Abstract

**Highlights:**

The study focuses on the design and comparison of capacitive pressure sensors using distinct biocompatible materials for in-stent blood pressure. The circular electrode design with crescent-shaped slots and a PDMS dielectric material demonstrated the highest sensitivity.

**What are the main findings?**

- The circular sensor design with two crescent-shaped slots, a 20 µm thick PDMS dielectric, achieved a sensitivity of 10.68 fF/mmHg.
- The resonant frequency shift showed an exceptionally linear relationship with blood pressure (Pearson’s correlation coefficient of −0.99986 and R-squared value of 0.99972).

**What is the implication of the main finding?**

- The high sensitivity and linear response of the sensor make it an ideal contender for precise, real-time pressure-monitoring applications.
- The optimized sensor design can be used in passive wireless pressure-sensing techniques for continuous health monitoring, particularly in detecting in-stent restenosis.

**Abstract:**

This research outlines the design of capacitive pressure sensors fabricated from three biocompatible materials, featuring both circular and square geometries. The sensors were structured with a dielectric layer positioned between gold-plated electrodes at the top and bottom. Their performance was assessed through simulations conducted with ANSYS Workbench. Of the various sensor configurations tested, the circular design that included two crescent-shaped slots and a 20 µm thick PDMS dielectric material demonstrated the highest sensitivity of 10.68 fF/mmHg. This study further investigated the relationship between resonant frequency shifts and arterial blood pressure, revealing an exceptionally linear response, as evidenced by a Pearson’s correlation coefficient of −0.99986 and an R-squared value of 0.99972. This confirmed the sensor’s applicability for obtaining precise blood pressure measurements. Additionally, a 3 × 30 mm cobalt–chromium (Co-Cr) stent was obtained, and its inductance was measured using an impedance analyzer.

## 1. Introduction

Coronary artery disease (CAD) is a condition that arises due to the narrowing of coronary arteries because of the accumulation of plaque on the arterial walls. This narrowing impedes the blood flow through the arteries, increasing the risk of heart attacks and strokes. The plaque, which is primarily composed of smooth cells such as macrophage cells, muscle cells, and various other materials such as cholesterol, sulphated glycosaminoglycan, collagen, and fibrin, contributes to the gradual constriction of the arteries [1]. Intravascular stents have emerged as a common intervention to address stenosis coronary arteries, serving as scaffolds to maintain arterial patency and restore blood flow. A stent implantation is the most preferable choice because of its high success rate and because it is the least invasive method [2]. However, the presence of metallic stents within arteries can trigger inflammation, leading to excessive tissue growth within the stent; this is known as in-stent restenosis [3]. The incidence of in-stent restenosis among patients with stents can be significant, with the rates reported to reach as high as 50% [4]. To mitigate this risk, medicine-coated drug-eluting stents that slow proliferation are commonly used to mitigate the issue of restenosis in the early stages post-implantation. However, concerns have been raised regarding the long-term safety of stents, as they may increase the risk of thrombosis and cases of subsequent heart attacks. Despite the availability of preventive measures, the identification of in-stent restenosis typically relies on invasive methods such as a duplex ultrasound and angiography, which are usually reserved for patients presenting with symptoms such as chest pain. As a result, there is a need for non-invasive and more accessible methods for detecting in-stent restenosis, particularly in asymptomatic patients [5]. Moreover, diabetic patients often lack chest pain sensation, even as arterial narrowing recurs, potentially leading to sudden and perilous health deteriorations. While X-ray-based inspection devices offer continuous metal stent monitoring, they are costly. Therefore, identifying alternative real-time monitoring approaches for both metal stents and blood pressure is crucial. Compared to conventional sensors, micro-electromechanical systems (MEMS)-based sensors offer several advantages, including high efficiency, small size, and affordability. These sensors combine miniature electrical and mechanical parts, improving system performance and lowering overall system expenses.

Many sensing techniques, including piezoelectric, capacitive, and piezoresistive sensing, are employed. Capacitive pressure sensors are favored for their high sensitivity, power efficiency, minimal temperature drift, and different designs, such as circular, square, and elliptical shapes [6]. The optimization of the design and the maximization of sensitivity while keeping the dimensions realizable and ensuring easy fabrication are some of the major challenges that have been reported in the literature. As the world becomes increasingly digitalized, the demand for new technologies has surged. MEMS have emerged as a transformative technology, offering a small, lightweight size; enhanced performance; and reliability across various sectors, such as the automotive and instrumentation sectors. MEMS sensors, which are significantly smaller than traditional sensors, are particularly adept at measuring pressures such as dynamic wall pressure and shear stress [7]. An LC circuit has been integrated with a capacitive triboelectric pressure sensor (CTPS) for real-time wireless sensing demonstrating a self-powered wireless pressure sensing system (SP-WPSS). Employing such a system provided the model with high sensitivity, fast response, and low detection limit. Efficient wireless monitoring over a long distance was achieved through impedance matching with a gas discharge tube. This was achieved by adjusting the resonant frequency as a function of pressure [8].

In another related research, the author integrated an SP-WSS with a triboelectric nano-generator (TENG), providing a combined functionality of energy harvesting, sensing, and communication of real-time operation. An LC circuit comprising an inductor coil and a capacitive pressure sensor lowers energy usage and permits signal transmission with a 40 m transmission range [9]. A self-powered nanofluidic pressure sensor has been designed to harness ionic movement through nanochannels, directly linking pressure changes to ionic current. This unique approach removes the need for an external power source while delivering high sensitivity, fast response, and long-term stability. With these advantages, the sensor shows great promise for real-time pressure monitoring in both medical and industrial settings [10].

To address the need for the wireless monitoring of restenosis, smart devices utilizing MEMS technology have been reported. These devices leverage a series inductor–capacitor (LC) resonant circuit to enable diverse transducer features, such as the wireless sensing of blood pressure using telemetry. The authors of another article introduced a helically shaped stent made up of stainless steel equipped with pressure sensors, where the electromagnetic coupling used inductive and capacitive elements to act as communication devices for restenosis detection. However, helical stents are unable to resolve re-narrowing issues when in-stent restenosis arises [11]. The imperative to detect in-stent restenosis non-invasively and rapidly has spurred advancements in smart stent technology, among other solutions [12]. Implantable sensing technology can be categorized into active and passive. An active system constitutes an electronic circuit and power sources to enhance functionality. However, the complexity varies with different packaging methods, and there are reliability issues with device linkages, making this type of system an unreliable choice for medical implant devices or within blood vessels due to associated lifetime constraints. In contrast, passive devices constitute an inductive coil excited externally with mutual coupling, and the power is supplied wirelessly for the sensing operations. These systems boast a simple structure [13]. Passive sensors work based on mutual coupling between the sensor coil and the external transmitter coil, overcoming the limitations of integrated power supplies. Such systems excel in wireless techniques due to favorable specifications and superior performance across a wide frequency band. Recent research has focused on developing highly efficient, low-powered, and reliable medically implanted devices that can offer high data rates [14]. Researchers have developed a pressure sensor using polyimide, integrated with an LC resonant circuit for wireless power transfer. However, the high resonant frequency of this sensor, which stems from compaction and high sensitivity, may cause cell disruption if implanted within the human body [15]. Gong et al. developed a pressure sensor with enhanced sensitivity using gold nanowires and a power-efficient system for obtaining pressure measurements [16]. Other researchers have proposed various sensor types with different levels of flexibility, power consumption, and biocompatibility. Recent improvements in capacitive pressure sensors now feature self-repairing, recyclable materials like poly (disulfide) polymers with special bonds and metal-catechol complexes. These materials have great sensitivity (up to 9.26 kPa^−1^) and fast response times. They also maintain consistent performance after self-healing and recycling, providing possibilities for sustainable and high-performance sensing systems [17]. Dielectric material selection is a crucial aspect, as implantable devices require biocompatible materials to be utilized in the design of the pressure sensors. The most widely used biocompatible and hydrophobic dielectric material is polydimethylsiloxane (PDMS). It offers suitable electrical and mechanical properties, which is why this elastomer is widely used in biomedical applications [18]. The authors of [18] briefly reviewed the wide application of PDMS in the design of micro-fluid, nanostructure-based biomedical applications, especially in implantable devices. Polyurethane is another dielectric material that is utilized by researchers for many biomedical applications, especially flexible and pressure sensors [19]. Recent advances have demonstrated MXene/cellulose nanofiber composite membrane-based nanofluidic pressure sensors that convert mechanical signals into electrical energy, enabling self-powered operation. These sensors have increased sensitivity and detection ranges, making them appropriate for designing portable and wearable devices [20]. There are several other biocompatible materials, but this specific application of in-stent restenosis measurements (correlated to a change in pressure due to plaque and fatty acid deposition) demands specific electrical and mechanical properties. One such dielectric material that has been widely used for biomedical applications is silicone rubber [21].

However, challenges persist regarding signal interference and a wireless power supply [22]. Passive devices eliminate the need for batteries and active circuitry, enabling compact designs suitable for implantation in small arteries such as the coronary artery [23]. The integration of a minimally invasive blood pressure-measuring device for continuous monitoring with a coronary stent can act as an early warning system for cardiac health. Wireless sensing technology has been widely used in biomedical applications. Telemetry and telemedicine have been heavily researched lately [24]. Traditionally, telemetry-based medical devices in the radio frequency (RF) range use an LC coupling resonant circuit, in which the frequency shifts with the physiological parameter of interest. Here, the change in capacitance due to arterial blood pressure is translated into a shift in the resonant frequency of the transmitter. The wireless determination of the resonant frequency enables the extraction of diagnostic information by analyzing the impedance of an antenna placed in the vicinity of the implanted device via electromagnetic coupling. In vascular applications, MEMS-based wireless sensors have gained traction [25]. One article found that for a capacitive sensor in combination with a gold-plated spiral inductor (2.6 mm × 1.6 mm), the size remained a concern, despite demonstrating effective performance [26]. In another research article, the authors proposed a 1.5 × 1.5 × 0.2 mm^3^ sensor that consisted of a 316L stainless steel chip micro-machined to form a cavity of 1 × 1 mm^2^ with a depth of 11–15 μm, which served as one of the capacitive electrodes. An Au–polyimide (PI) multilayer diaphragm hermetically sealed the cavity at atmospheric pressure, acting as the other flexible capacitive electrode that changes with applied external pressure [27].

For pressure sensing, various sandwiched microstructures have also been reported. In these microstructures, a microstructure inotropic film is sandwiched between two capacitor electrodes, resulting in significant capacitance changes. A shift in the resonant frequency of the tank circuit was wirelessly acquired using a phase dip technique. For an applied pressure range of 0–250 mmHg, a frequency shift from 429 MHz to 227 MHz was observed [28]. In most of the reported capacitive pressure-sensing techniques used to measure arterial blood pressure for in-stent restenosis monitoring, the reported sensitivity still needs to be improved [29]. In this study, a novel design is proposed for the capacitive pressure sensor previously developed by the above researchers to enhance its detection sensitivity.

In this work, two different types of pressure capacitive sensors (one with circular and the other with square-shaped electrodes) were designed. Three different types of biocompatible materials, namely PDMS, polyurethane, and silicone rubber, were selected as the dielectric materials for the proposed pressure sensors. These materials provided flexibility and enhanced the sensor’s sensitivity. Crescents were cut into both the structures for all three materials; hence, a total of eighteen sensors comprising six different sensor structures and three different materials were designed. The pressure versus deflection was recorded, and finally, due to the deflection caused by applied pressure, the corresponding capacitance values for each sensor are graphically presented. The optimization of dimensions and structures was accomplished in the simulation study. The sensitivity of the sensors was compared, and the most suitable structure and material, as well as the sensor offering the maximum sensitivity, were determined. The article organization constitutes the following: Section 1 provides the principles of operation, Section 2 explains the sensor design and biomaterial properties, Section 3 presents the results and discussion, and Section 4 provides the conclusion of this research.

## 2. Materials and Methods

**Figure 1:**
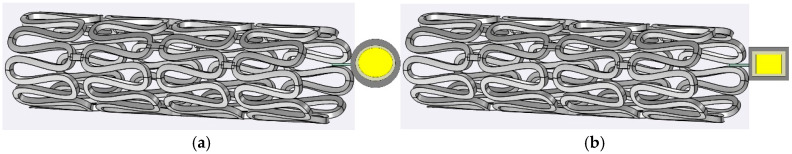
In-stent pressure capacitive sensors: (**a**) a circular-plate capacitive sensor; (**b**) a square-plate capacitive sensor. Central part golden/yellow color represent gold electrodes, and the grey color is representing sensing layer of polymer.

A finite element analysis facilitated the evaluation of changes in the capacitance value for the applied pressure range, guiding the design of the inductor coil for optimal performance. This study underscores the importance of resonant frequency selection and inductor design considerations for wireless pressure sensors in biomedical applications. Various MEMS sensors and structural designs were explored to design sensors that observe deflection with the applied pressure. The designed sensors are to be integrated upstream of the implanted stent. Figure 1a shows the schematic diagram of the circular sensor design, and Figure 1b shows the schematic diagram of the square sensor design, both intended for installation at the tip of the stent structure.

With the blood flow through the implanted stent sensor system, the top plate of the sensor deflects according to the upstream pressure build-up, causing a change in the sensor’s capacitance. As the plaque deposition increases, the pressure build-up also increases proportionally; hence, the change in capacitance can be used for the continuous monitoring of restenosis. This study considered six novel designs for the deflective plate: a square plate with no slots, a square plate with four I-shaped slots on the sides, a square plate with four L-shaped slots in the corners, a circular plate without slots, a circular plate with two crescent cuts, and a circular plate with four crescent-shaped cuts.

The change in capacitance can be obtained using an external transmitter circuit that employs electromagnetic coupling. The static inductance value (L) due to the fixed stent structure in series with a variable capacitor (pressure sensor) that changes with an increase in arterial pressure due to restenosis results in a shift in the resonant frequency. The change in capacitance correlated positively with the change in arterial pressure, which, in turn, can be easily reflected in terms of a resonant frequency shift in the transmitted signal due to electromagnetic coupling. With the designed number of spiral loops attached together to form a stent structure, L (static) varies from structure to structure, which may lead to a change in the base resonant frequency. This is given by the following:

(1) $$\omega_{0}=2\pi f_{0}=\frac{1}{\sqrt{L_{s}C_{s}}}$$

In this work, the focus was to optimize the dimensions and the selection of the most suitable biocompatible material for a wireless smart capacitive pressure sensor. The most sensitive design was selected based on performance and sensitivity, and it was then optimized and analyzed in comparison to a typical square plate for the continuous monitoring of in-stent restenosis.

To solve the challenges related to power supply and sensor integration, it is proposed to employ passive wireless energy harvesting through RF or inductive powering, enabling the capacitive pressure sensor to function without batteries by drawing power from external sources. The capacitance change in the sensor is converted into an electrical signal through a capacitive-to-voltage converter, followed by signal conditioning using operational amplifiers and analog to digital converters. The processed signal can be wirelessly transmitted using a low-power communication module such as Bluetooth Low Energy (BLE) or RF, facilitating continuous and real-time blood pressure monitoring. This methodology will support the development of a fully integrated, wireless, and energy-efficient system for in-stent applications.

### 2.1. Sensor Design and Biomaterial Properties

#### 2.1.1. Sensor Structure

**Figure 2:**
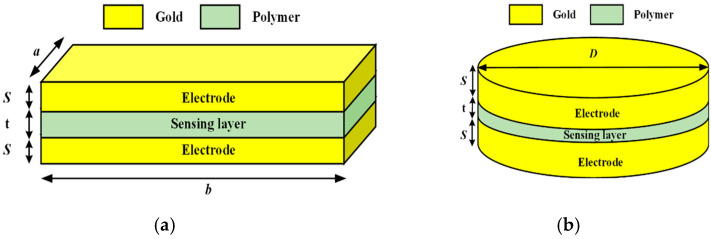
Schematic diagram of sensors: (**a**) a square parallel plate; (**b**) a circular parallel plate.

In this study, capacitive sensors are modeled employing ANSYS Maxwell 3D Version 2022, R2 software (ANSYS Inc., Canonsburg, PA, USA). It is a useful instrument for analyzing electromechanical and magnetic fields in complex 3D geometries. Furthermore, this tool provides accurate capacitance measurements and enhanced sensor performance. The parallel-plate sensors, one with circular and another with square-shaped gold-plated electrodes, are first simulated. Gold was selected as the electrode material due to its excellent biocompatibility, non-corrosive nature, high conductivity, and mechanical stability, which are crucial for long-term performance in biomedical applications. Electrodes for a circular plate with a diameter of 1 mm and a thickness of 20 µm were prepared. One of the electrodes was attached to the stent on which the sensing dielectric layer was deposited, and on the sensing layer, the second electrode of gold material was formed in order to realize a parallel-plate capacitive sensor in the software. A dielectric material sandwiched between the two electrodes resulted in a separation of 20 µm between the two electrodes of the sensor. For the square-shaped electrode, an edge length of 0.5 mm and a thickness of 20 µm was used for the gold-plated electrode, and a dielectric material of the same dimensions was sandwiched between the plates. Figure 2a shows the schematic diagram of the square-shaped electrode design with dimensions, and Figure 2b shows the schematic diagram of the circular-shaped electrode design with dimensions. Both these images are generated using Microsoft Visio 2023 Version 2024.

**Figure 3:**
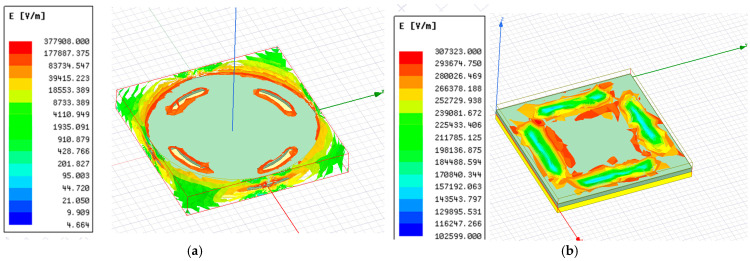
Electric field intensity for a solid electrode with four cuts: (**a**) a circular plate capacitive sensor; (**b**) a square plate capacitive sensor.

The materials were assigned to the various geometries using the library of the software. The sensing materials (PDMS, polyurethane, and silicone rubber) were added to the library using the dielectric constant and bulk conductivity. The creation region was used to provide an electrostatic shield to the sensor. Triangular meshing was used for simulation. The electrodes were excited with a voltage (+1 V for the upper electrode and GND for the bottom electrode), and the dielectric material was allocated after selecting a specific dielectric constant and bulk conductivity; then, the base value of the capacitive sensors was evaluated for each dielectric material. The capacitive sensors implemented by other researchers have typically used square-shaped parallel-plate electrodes. The base electrode is kept fixed while the top electrode is deflected with the applied pressure range. The generic formula for the parallel-plate capacitive sensor is given by Equation (2) [30].

(2) $$C=A\frac{\varepsilon_{o}\varepsilon_{r}}{d}$$

where *A* is the common area of the electrodes, *d* is the separation between the plates, *ε_o_* = 8.85 × 10^−12^ F/m, and *ε_r_* is the dielectric constant of the medium between the electrodes. Figure 3a shows the electric field distribution for the circular-shaped electrode with four cuts, and Figure 3b shows the electric field distribution for the solid square electrode with four cuts. It is observed that the field intensity near the electrodes was maximized.

In further analysis, slots were cut into the circular and square structures of the sensing layer. For the circular dielectric layer, the slots were formed as crescent shapes (with two or four cuts). In the square dielectric structure, the cuts were made in the shape of an I (parallel to the edges) or an L (at the corners) to develop different structures, with the goal of optimizing the dimensions and overall sensitivity. A total of 18 sensors were simulated, and their performance was evaluated to identify the most sensitive capacitive pressure sensor out of the proposed structures and biocompatible dielectric materials. Table 1 presents the simulation parameters of the designed sensors in detail.

#### 2.1.2. Biocompatible Material Selection

Three biocompatible materials, namely PDMS, polyurethane, and silicone rubber, were selected for the dielectric layer of the proposed sensors. All three materials offer excellent mechanical and electrical properties and are biocompatible, highly flexible, compressible, hydrophobic, and stable, making them highly suitable for implantable devices such as in-stent smart capacitive pressure sensors.

1. Polydimethylsiloxane (PDMS)

PDMS is widely used in biomedical applications, and it has been explored for medical implants because it offers good osseointegration [31]. Despite its biocompatibility, it is less reactive, and its microscale features facilitate the bonding of the implant with body organs, especially bones. Glass and other polymers represent a substantial labor cost and require complex machining processes due to their rigidity, whereas PDMS is an elastomer with excellent thermal and electrical properties and is a hyper-elastic material that can tolerate large amounts of deflection before rupture. Additionally, PDMS is excellent at mimicking blood vessels [32]. It is one of the most suitable materials for biomedical applications. In this work, this material was selected due to its extraordinary properties to act as a dielectric layer in the capacitive pressure sensor.

Several aspects contribute to the optimized capacitive pressure sensor’s better results in terms of inductance, frequency range, and sensitivity. The addition of PDMS, recognized for its high dielectric constant, flexibility, and biocompatibility, improves sensitivity and conformance to the artery wall. The round electrode design reduces edge effects by equally dispersing stress, resulting in improved mechanical stability. Miniaturizing the electrode (1 mm diameter, 20 µm thickness) reduces parasitic capacitance and increases frequency range. Structural improvement and stability of the resonance frequency improve the efficiency of wireless monitoring. These combined developments make the sensor ideal for continuous in-stent arterial pressure monitoring.

The circular PDMS capacitive pressure sensor’s environmental stability and long-term performance have been extensively evaluated for dependable operation in simulated essential settings. PDMS, known for its better biocompatibility and resistance to chemical degradation, sustains its mechanical characteristics and dielectric constant throughout time. Its minimal water absorption eliminates moisture-induced capacitance variations, and long-term tests in high humidity reveal insignificant performance changes. The sensor also maintains structural integrity and electrical qualities in the face of cyclic pressure and temperature variations, with low hysteresis and high repeatability. These features ensure that the circular PDMS capacitive pressure sensor retains its excellent sensitivity and accuracy, making it suitable for continuous arterial pressure monitoring.

2. Polyurethane Rubber

Polyurethane rubber is a bio- and blood-compatible material that is used for the development of everything from simple biomedical devices, such as catheters, to the most complex device, an artificial heart [33]. It is highly durable, elastic, fatigue-resistant, and acceptable within the body, making it suitable for use in implantable devices. It behaves like a natural tissue due to its acceptability within the body during healing processes. It has been used in the design of many functional devices for biomedical applications [34]. Hence, polyurethane rubber was the second dielectric material selected for the analysis in this work.

3. Silicone Rubber

Silicone rubber is an elastomer that is highly durable and biocompatible. It has a wide range of thermal and chemical resistance, enabling it to tolerate hot tissue attacks; it also acts as an insulator for electronic implants such as pacemakers, etc. It is an elastic material that can retain its mechanical properties within a very wide range of temperatures [35]. This material is very suitable for biomedical applications and the design of medical implant devices. Thus, it was selected as the third dielectric material for the design of sensors.

The proposed design is unique in that it examines the effects of PDMS, polyurethane, and silicone rubber on sensor performance independently. A systematic assessment of each material’s flexibility and durability was shown by getting deformation vs. pressure and capacitance vs. pressure graphs. In this study it is observed that PDMS has the maximum sensitivity due to its higher elasticity, whereas polyurethane establishes a compromise between flexibility and mechanical strength, and silicone rubber has excellent stability. Structural improvements, such as a multi-layered composite construction and micro-structuring of dielectric layers, improve sensitivity and frequency response. Compared to earlier studies on single-material sensors, the presented design in this study provides a full performance evaluation, streamlines the fabrication process, and lowers costs, emphasizing the unique benefits of these materials.

The referenced paper [27] makes use of polycrystalline silicon (Poly-Si) with square and circular plates that have straight, L-shaped, and crescent-shaped slots with a plate gap of 10 μm. In contrast, the proposed design investigates numerous dielectric materials, including PDMS, polyurethane rubber, and silicone rubber, using modified plate designs with similar slot layouts. The use of PDMS in this study allows for more deflection and elasticity, which addresses the mechanical constraints of Poly-Si in the cited work. Furthermore, this study includes a detailed comparison of capacitance versus pressure for each material, providing greater information about the impact of sensor design and slot selection on total sensor performance. Table 2 compares the results of the proposed design with the existing study.

Despite major changes in material characteristics and structural designs between the proposed and referenced designs, the linearity of capacitance changes with respect to pressure is nearly the same for both sensor designs.

## 3. Results and Discussion

### 3.1. Inductance Measurement of the Stent

**Figure 4:**
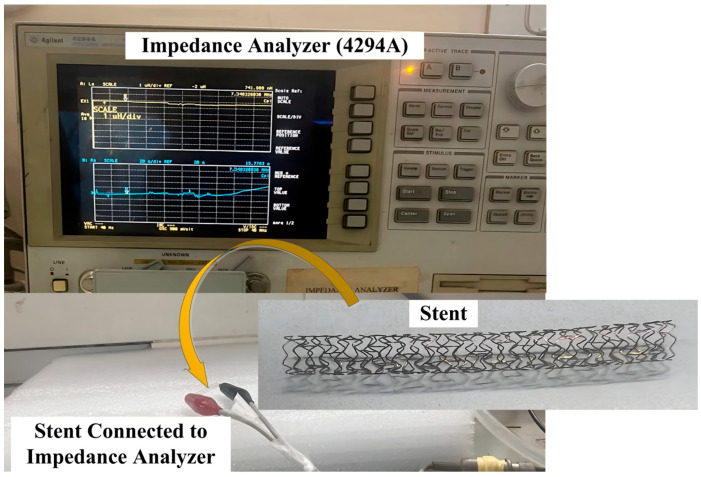
Electrical characterization of stent using impedance analyzer.

A BX-type cobalt–chromium (Co-Cr) stent with a length of 30 mm and a diameter of 3 mm was procured from Biotronik Medical Devices India Pvt. Ltd. (New Delhi, India), Holy Family Hospital, New Delhi, India. The stent was inflated using a stent inflator by applying a nominal pressure of 10 atm using a sirolimus-eluting coronary stent ballooning system. The fully inflated stent was used for electrical characterization. Figure 4 shows the connection of both ends of the stent to an impedance analyzer (4294A) using alligator clips.

The inductance value of the stent was measured in the frequency range of 100 kHz–110 MHz using an impedance analyzer. The inductance values of 68 nH at 110 MHz and 1039 nH at 100 KHz were obtained. The value of L (inductance) used for the calculation of the resonant frequency using Equation (1) was 927 nH, calculated at 110 MHz. A change in the capacitance value (Cs) with an increase in arterial blood pressure (60 mmHg–200 mmHg) was observed for the designed capacitive pressure sensors (circular structure with two crescent cuts). The shift in capacitance resulted in a shift in the resonant frequency, as described in a later section.

### 3.2. Capacitive Response of the Sensor

The capacitance values in response to the pressure applied were evaluated for two different parallel-plate capacitive pressure sensors with three different dielectric materials. The first structure, which used solid, circular-shaped electrodes with a diameter of 1 mm and a thickness of 20 µm and the three dielectric materials (namely PDMS, polyurethane rubber, and silicone rubber), was analyzed for an applied pressure of 60 mmHg to 200 mmHg.

**Figure 5:**
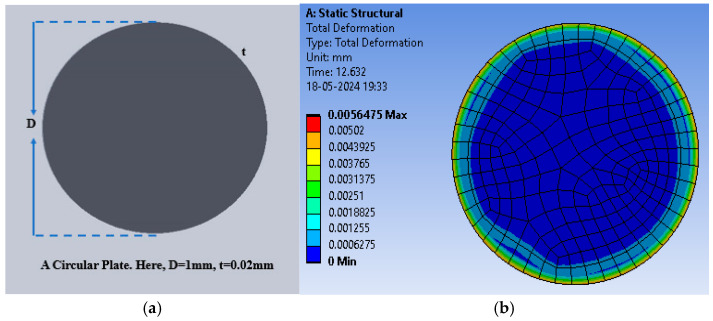
(**a**) A circular plate capacitive sensor; (**b**) total deflection in the circular plate sensor for the pressure range of 60 mmHg to 200 mmHg with PDMS as the dielectric material.

Gold electrodes with a thickness of 20 µm deposited on both sides of the dielectric material were used as the plates of the capacitive sensor. Figure 5a shows the schematic diagram of the circular plate capacitive sensor structure. The simulation study was conducted on a PC (Intel i5 8th Generation, 8GB RAM, 1.60 GHz, 1TB disk size). Figure 5b shows the color-coded animation of the deflection (in mm) for the pressure range applied to the sensor with PDMS as the dielectric material, generated using the ANSYS Workbench platform. ANSYS Workbench is an integrated simulation platform that provides a comprehensive environment for performing structural, thermal, fluid, and electromagnetic analyses.

For the solid circular structure, three different dielectric materials were selected one by one to analyze the deflection (in mm) with the selected pressure range (60–200 mmHg) in steps of 10 mmHg. The selected materials are biocompatible, hydrophobic, and widely used in implantable devices. The electrical and mechanical properties of the dielectric materials were inputted into the simulation software library for further analysis. Table 3 presents the values of bulk conductivity and dielectric constants of the selected materials.

**Figure 6:**
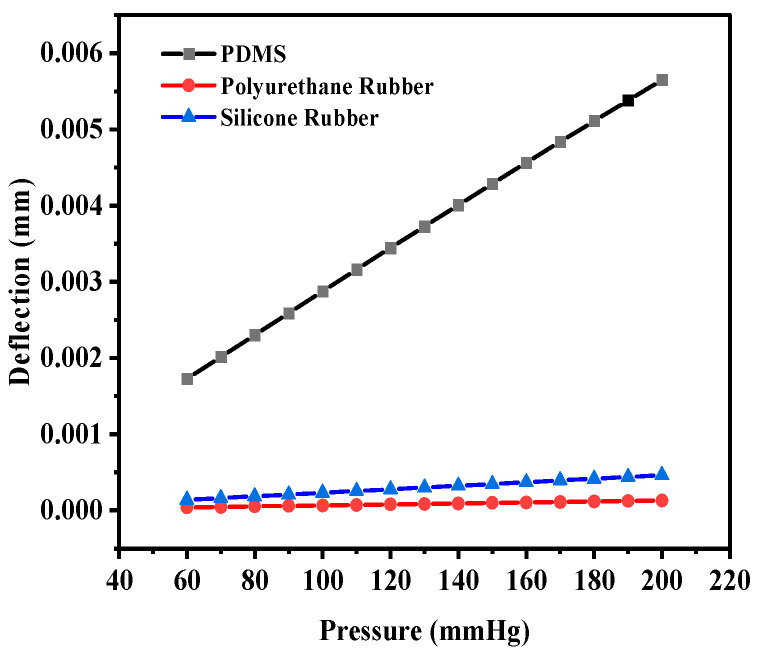
Deflection versus pressure for solid circular-shaped structure.

Initially, PDMS was used as the dielectric material to calculate the deflection (in mm) for the selected range of applied pressure; thereafter, the polyurethane rubber was used, and lastly, the silicone rubber was used. The circular structure (without any slots) offered the maximum deflection of 5.648 × 10^−^^3^ mm for PDMS, whereas the deflection was 1.3000 × 10^−^^4^ mm and 4.6210 × 10^−^^4^ mm for polyurethane rubber and silicone rubber, respectively. The deflection corresponding to the applied pressure was obtained using the ANSYS Workbench 2024R1 software. Figure 6 shows the plot of deflection versus pressure for the dielectric materials. Among the three selected materials, PDMS offered the maximum deflection for the selected range of pressure.

The main goal of the parallel-plate pressure sensor design was to develop a variable capacitor with applied pressure. The capacitance values were evaluated using the ANSYS EM18.0 Electronics software. The pressure was uniformly applied to the top electrode of the sensor’s surface, and the deflection was observed. Table 4 presents the theoretical and simulated capacitance values corresponding to the applied pressure for the solid square-shaped and solid circular electrode sensors with a PDMS sensing layer. It can be clearly seen from the table that the capacitance values obtained in the simulation study confirm the theoretical values obtained using Equation (2).

**Figure 7:**
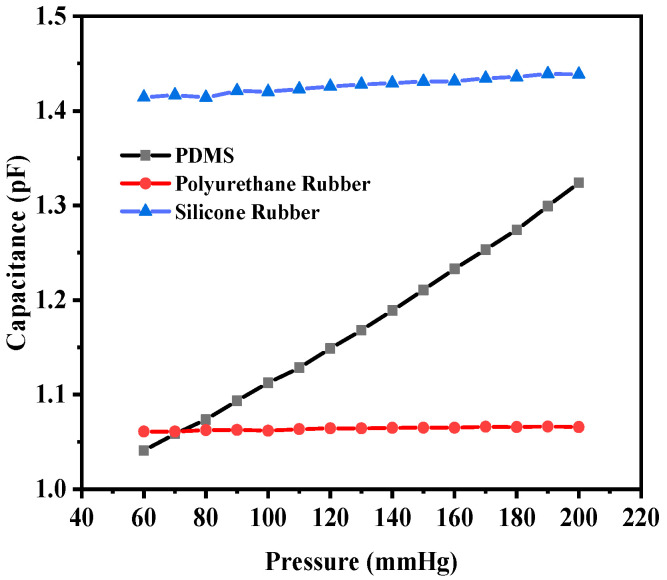
Response of circular capacitive sensor.

The corresponding capacitance value of the sensor in response to the deflection due to the applied pressure was obtained. The capacitance value without any deflection, also known as the base capacitance, was 0.95063 pF, 1.0585 pF, and 1.4055 pF for PDMS, polyurethane rubber, and silicone rubber, respectively. Since PDMS exhibited the maximum deflection, the capacitance at the maximum deflection was 1.324 pF, whereas for polyurethane rubber and silicone rubber, the values were 1.0656 pF and 1.4386 pF, respectively. While silicone rubber had the highest dielectric constant, resulting in a higher absolute capacitance, PDMS demonstrated the steepest capacitance–pressure response, as observed in Figure 7. This indicates that PDMS offers the highest sensitivity, making it the most suitable material for pressure-sensing applications. Figure 7 shows the plot of capacitance versus pressure for the sensor. It can be observed that the capacitance versus pressure curve varied linearly for PDMS as the dielectric material, whereas for the other two dielectric materials, the variation was close to linear. The graph shows the linear fitted curves created using the OriginPro 2024 software (OriginLab Corporation, Northampton, MA, USA). Origin Pro software is a data analysis and graphing tool widely used in scientific research to visualize, analyze, and interpret experimental data.

The sensitivity values of the sensor for all three selected materials were evaluated as the slope of the capacitance-versus-pressure curve for the selected range of pressure. The sensitivity value was calculated in fF/mmHg using Equation (3).

(3) $$Sensitivity=\frac{C_{max}-C_{min}}{P_{max}-P_{min}}$$

where Cmax and Cmin are the capacitance values in response to the Pmax and Pmin, respectively. The range of pressure considered for the analysis was 60 mmHg (Pmin) to 200 mmHg (Pmax), with a step change of 10 mmHg. The solid circular electrode sensor with PDMS as the dielectric material offered a maximum sensitivity of 2.01 fF/mmHg, whereas the sensitivity values for polyurethane rubber and silicone rubber were 3.8785 × 10^−^^2^ fF/mmHg and 1.85429 × 10^−^^1^ fF/mmHg, respectively.

The sensitivity observed for the solid circular structure can be improved if the deflection with pressure increases for each step of the rise in pressure.

**Figure 8:**
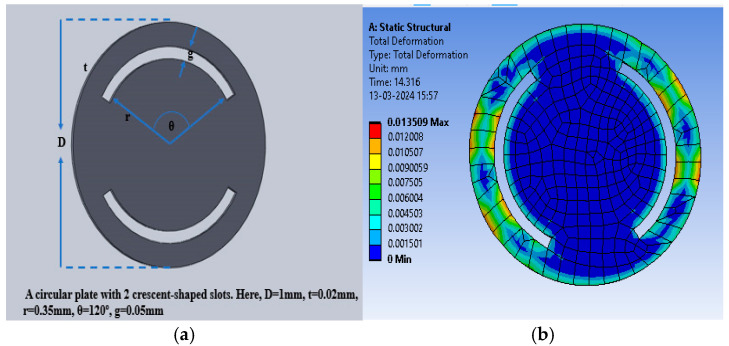
(**a**) Circular electrode with two crescents; (**b**) total deflection in the circular plate sensor with two crescents for a pressure range of 60 mmHg to 200 mmHg, with PDMS as the dielectric material.

The slots were cut in the selected dielectric material in the form of crescents to enhance the deflection and thus the capacitance value to enhance the sensitivity. Figure 8a shows the crescents cut at a radial distance of 0.35 mm for an angular range of 120° (60° to the left and right from the vertical plane of the sensor), with a gap of 0.05 mm. Figure 8b shows the color intensity-based deflection corresponding to the applied pressure.

**Figure 9:**
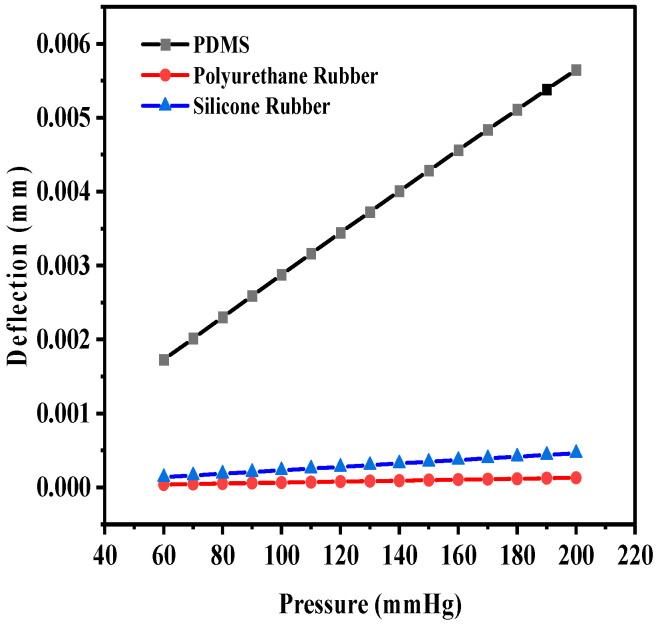
Deflection versus pressure for solid circular-shaped structure with two crescents.

The maximum deflection for PDMS as the dielectric material was 1.35 × 10^−^^2^ mm, whereas the maximum deflections observed were 4.56 × 10^−^^4^ mm and 1.8219 × 10^−^^3^ mm for polyurethane rubber and silicone rubber, respectively. With two crescents, the maximum deflection was also observed for PDMS in comparison to the other two dielectric materials. Figure 9 shows the plot of deflection (mm) versus pressure (mmHg) for the sensor structure (circular with two crescents).

**Figure 10:**
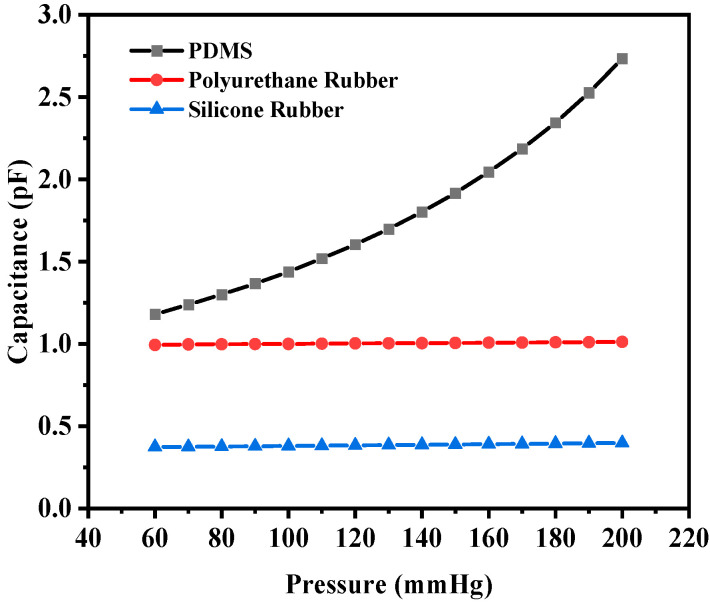
Response of circular capacitive pressure sensor (with two crescents).

The corresponding maximum capacitance value with the maximum deflection due to the applied pressure for PDMS, polyurethane rubber, and silicone rubber was 2.7334 pF, 1.0127 pF, and 0.3987 pF, respectively. The base value (without deflection) of the capacitive sensor was 0.89248 pF, 0.98935 pF, and 0.36336 pF, respectively. Figure 10 shows the plot of overall capacitance variation with applied pressure for the structure (circular with two crescents).

**Figure 11:**
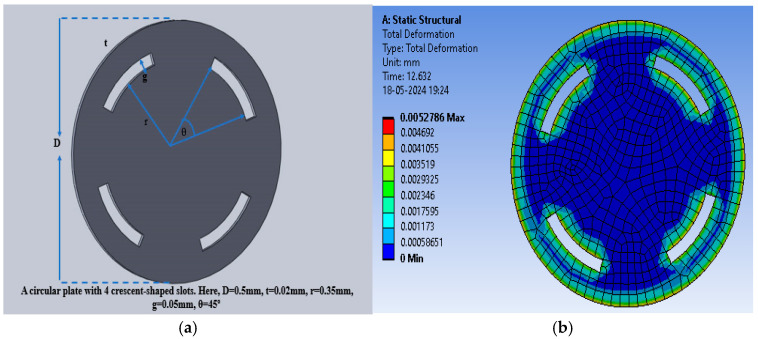
(**a**) Circular electrode with four crescents; (**b**) total deflection in circular plate sensor with four crescents for a pressure range of 60 mmHg to 200 mmHg, with PDMS as the dielectric material.

The sensitivity of the modified sensor was calculated using Equation (5). The PDMS-based circular sensor with two crescents offered a maximum sensitivity of 10.68 fF/mmHg, whereas the sensitivity values for polyurethane rubber and silicone rubber were 1.19443 × 10^−^^1^ fF/mmHg and 1.7994 × 10^−^^1^ fF/mmHg, respectively. Figure 11a shows the increase in the number of crescents to four in a circular pattern, maintaining the same radial distance and gaps, with a 45° angular separation, to enhance deflection and sensitivity. Figure 11b shows an animation of the color-coded deflection corresponding to the applied pressure.

**Figure 12:**
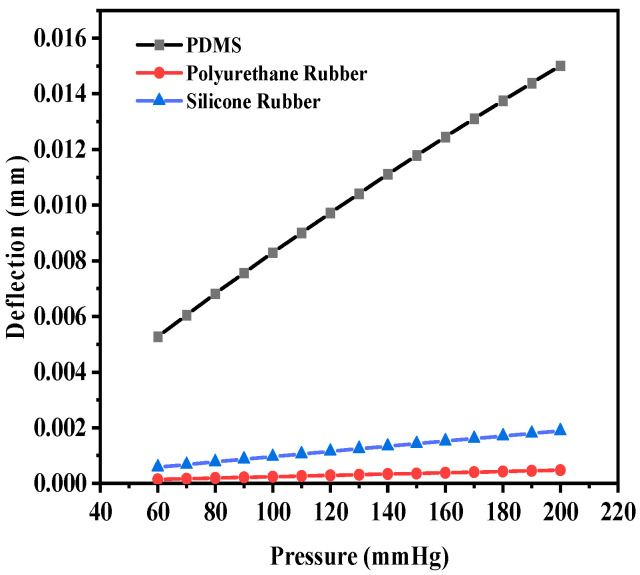
Deflection versus pressure for solid circular-shaped structure with four crescents.

Figure 12 shows the deflection versus pressure plot for the selected dielectric material in a similar manner. The maximum deflection values for PDMS, polyurethane, and silicone rubber were observed to be 1.5013 × 10^−^^2^ mm, 4.76 × 10^−^^4^ mm, and 1.89 × 10^−^^3^ mm, respectively. PDMS is spongier in comparison to the other two dielectric materials, which resulted in an increase in the maximum deflection. It was observed that for a dielectric material with a thickness of 20 µm, the cutting of four crescents did not result in a significant increase in the deflection compared to the structure with two crescents.

**Figure 13:**
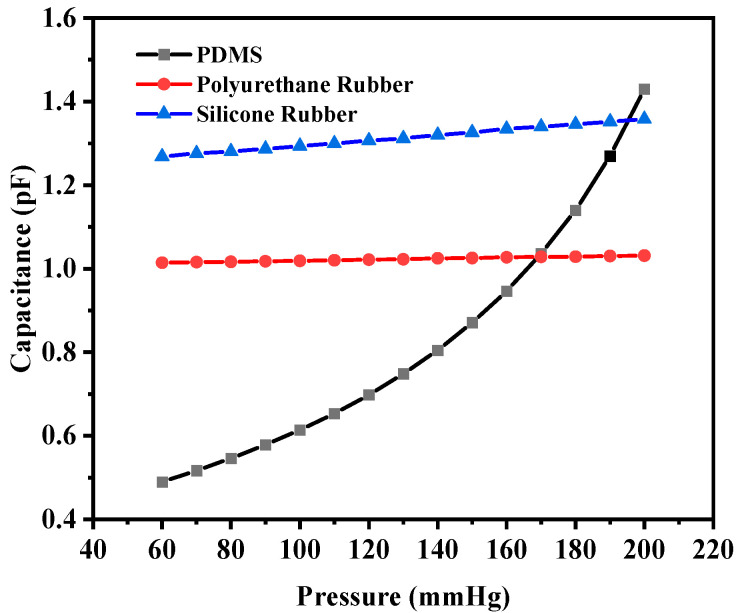
Response of circular capacitive pressure sensor (with four crescents).

The capacitance base values of this structure (without any deflection) were 0.36423 pF, 1.0072 pF, and 1.2321 pF for PDMS, polyurethane rubber, and silicone rubber, respectively. The base value of the capacitors increased in comparison with the previous structure for polyurethane rubber and silicone rubber because the dielectric constants were higher in comparison to that of PDMS. The capacitance values corresponding to the maximum deflection were 1.4302 pF, 1.0314 pF, and 1.3582 pF, respectively. Figure 13 shows the plot of capacitance variation corresponding to the change in pressure.

The sensitivity values of the four-crescent sensors were 6.23 fF/mmHg, 1.24429 × 10^−^^1^ fF/mmHg, and 6.4607 × 10^−^^1^ fF/mmHg, respectively. The overall sensitivity value for PDMS was decreased when increasing the number of crescents due to a reduction in the capacitance, whereas the sensitivity of the sensors with polyurethane rubber or silicone rubber increased because the change in the capacitance was higher (the dielectric constant of PDMS was lower than that of the other two materials). The sensor with more crescents was not favorable due to the loss of dielectric material and the reduction in the common area between the electrodes.

**Figure 14:**
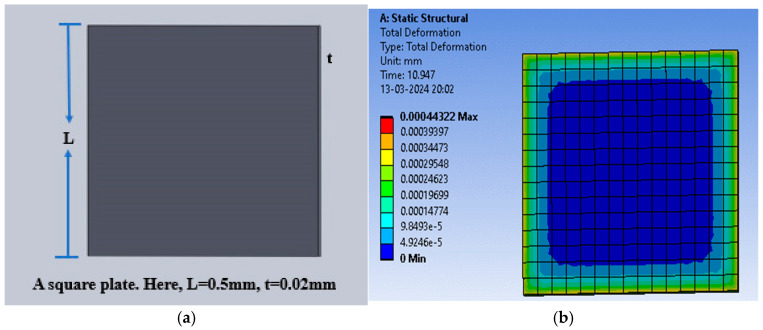
(**a**) Square electrode sensor; (**b**) total deflection in square plate sensor for a pressure range of 60 mmHg to 200 mmHg, with PDMS as the dielectric material.

One more structure with square-shaped electrodes was designed for capacitive pressure sensing. The enhancement in the sensitivity of the sensor was explored for three dielectric materials, namely PDMS, polyurethane rubber, and silicone rubber, and slots were cut to increase the maximum deflection and thus the sensitivity. Firstly, the solid square-shaped gold electrodes were deposited for the selected dielectric constants. Figure 14a shows the schematic diagram indicating the square electrode with an edge length of 0.5 mm and a thickness of 0.02 mm, and Figure 14b shows an animation of the deflection with the PDMS dielectric under the applied pressure range of 60 mmHg to 200 mmHg.

**Figure 15:**
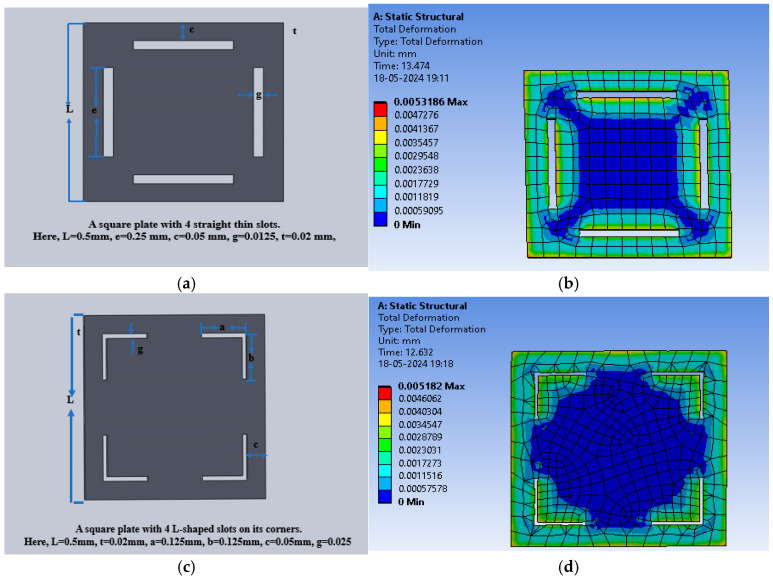
(**a**) Square dielectric material with four I-slots; (**b**) total deflection in the square plate sensor with four I-slots for a pressure range of 60 mmHg to 200 mmHg, with PDMS as the dielectric material; (**c**) square dielectric material with four L-slots; (**d**) total deflection in the square plate sensor with four L-slots for a pressure range of 60 mmHg to 200 mmHg, with PDMS as the dielectric material.

The square-shaped electrode was slotted with four I-shaped cuts or four L-shaped cuts to modify the structure and analysis of the sensitivity of the variable capacitor with the applied pressure. Figure 15a shows the slotted structure with four I-shaped cuts using PDMS as the dielectric material, and Figure 15b shows the color-coded animation of the deflection under the applied pressure range for the same structure. Further, Figure 15c presents the slotted structure with four L-shaped cuts using PDMS as the dielectric material, and Figure 15d shows the color-coded animation of the deflection under the applied pressure range for that structure.

**Figure 16:**
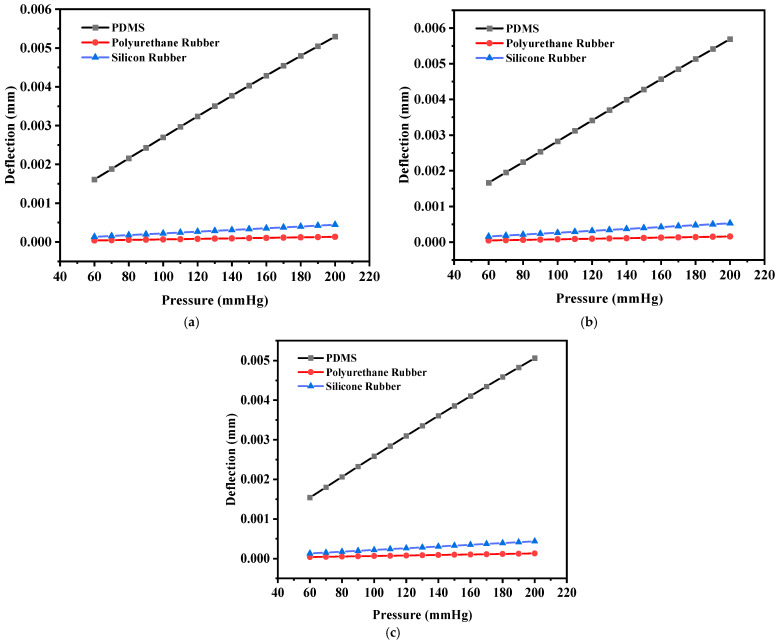
(**a**) Deflection versus pressure for the solid square-shaped structure; (**b**) deflection versus pressure for the solid square-shaped structure with four I-shaped slots; (**c**) deflection versus pressure for the solid square-shaped structure with four L-shaped slots.

Figure 16a shows the plot of deflection for all three-square structures (solid and slotted) against the applied pressure using PDMS as the dielectric material, and Figure 16b,c show the corresponding plots using polyurethane rubber and silicone rubber, respectively. The PDMS-based sensor showed an increase in the maximum deflection for small slots (four I-shaped slots); however, the maximum deflection decreased when L-shaped slots were cut due to loss of dielectric material.

Table 5 presents the parametric values, including the maximum deflection, base capacitance, and capacitance value due to the maximum deflection, for the designed capacitive sensors.

**Figure 17:**
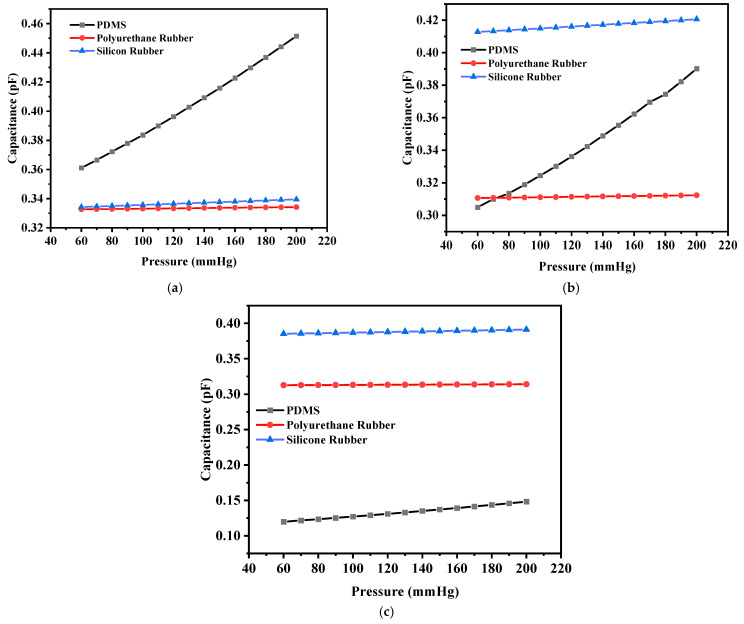
(**a**) Capacitance versus pressure for the solid square-shaped structure; (**b**) capacitance versus pressure for the solid square-shaped structure with four I-shaped slots; (**c**) capacitance versus pressure for the solid square-shaped structure with four L-shaped slots.

The capacitance versus pressure was also plotted in a similar fashion as for the circular structures. Figure 17a shows the plot for the solid square-shaped structure, and Figure 17b,c show the plots for the structures with four I-shaped cuts and four L-shaped cuts, respectively. It can be seen from Table 5 that despite the maximum deflection being lower in the case of silicone rubber, the corresponding capacitance value was higher than those of the other two dielectric materials because the dielectric constant of silicone rubber is greater.

The sensitivities of the designed square-shaped electrodes were calculated from the plots. It was evident that the sensitivity values obtained for the square-shaped electrodes with PDMS were higher than for the other two cases. Table 6 presents the sensitivity values of all the proposed sensor structures.

Two different structures were explored to analyze the sensitivity towards the applied pressure for the continuous monitoring of the restenosis process. The designs were modified, and the dimensions were optimized with slots cut into the structures. This study represents an attempt to optimize the sensor structure for a specific application with the maximum possible sensitivity using three biocompatible materials (PDMS, polyurethane rubber, and silicone rubber). It was concluded that the pressure sensor consisting of circular-shaped electrodes with two crescents offered the maximum sensitivity (10.68 fF/mmHg) for the pressure range of 60 mmHg to 200 mmHg.

### 3.3. Shift in Resonant Frequency

**Figure 18:**
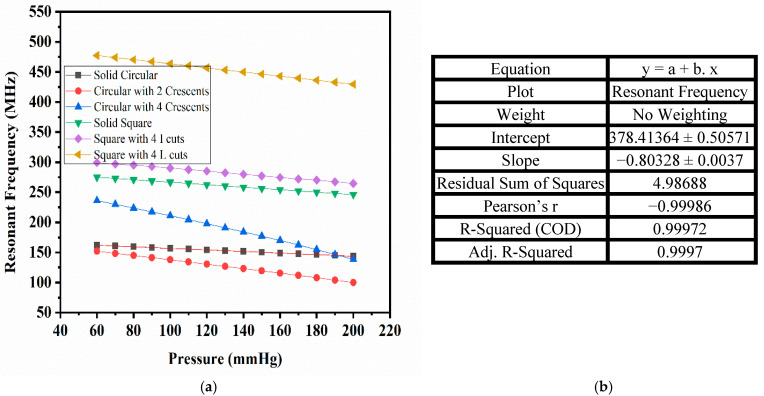
(**a**) Resonant frequency vs. pressure for designed sensors; (**b**) tabular representation of linear fit parameters for the circular electrode with two crescents (PDMS).

The changes in the capacitance values of the proposed sensors with the PDMS material for the pressure range of 60 mmHg to 200 mmHg are plotted in the previous section. Figure 18a shows the plot of the corresponding resonant frequency, calculated using Equation (1), for all the designed sensors with PDMS as the dielectric material over the selected frequency range. The maximum sensitivity was achieved for circular-shaped electrodes with two crescents designed using PDMS, and the change in the resonant frequency decreased linearly with an increase in the arterial blood pressure. Figure 18b shows the correlation between the shift in the resonant frequency and the applied pressure, obtained using a linear fit equation, with parameters such as Pearson’s coefficient and R-squared values being very close to unity. The response of this sensor was highly linear.

Table 7 presents a comparative analysis of previous research and the proposed capacitive pressure sensor system for in-stent arterial blood pressure measurements. The table highlights key parameters such as the sensor shape, material composition, inductance, frequency range, and sensitivity, showcasing the advancements in the proposed design. The proposed circular sensor structure offers higher sensitivity, optimized dimensions, and improved compatibility for in-stent applications.

## 4. Conclusions

This study demonstrated the design and optimization of parallel-plate capacitive pressure sensors for monitoring of in-stent restenosis. Two electrode configurations, circular and square, were designed using three different dielectric materials: PDMS, polyurethane rubber, and silicone rubber. The objective was to identify an optimal sensor structure and material combination that would provide maximum sensitivity for the early detection of restenosis. A stent (3 mm in diameter, 30 mm in length) was procured, and electrical characterization was conducted using an impedance analyzer. A total of 18 sensor designs were simulated, all featuring gold-plated electrodes with a thickness of 20 µm, and the effects of slots cut into the electrode structure were analyzed in relation to the sensor’s sensitivity. The results revealed that the circular electrode design with a diameter of 1 mm, a thickness of 20 µm, and two crescent-shaped slots at a radial distance of 0.35 mm and an angle of 120° exhibited the highest sensitivity. Among the dielectric materials tested, PDMS was found to be the most suitable material for the sensor design, particularly within the applied pressure range of 60 mmHg to 200 mmHg. The resonant frequency shift over the selected pressure range demonstrated a highly linear response. In conclusion, the sensor design derived from this study proved to be highly sensitive, making it a promising contender for futuristic use in arterial blood pressure measurements using passive wireless pressure-sensing techniques.

## Figures and Tables

**Table 1:** Simulation parameters.

Sensor	Parameter	Value
Circular	Electrode diameter (D)	1 mm
	Electrode thickness (S)	20 µm
	Sensing layer thickness (t)	20 µm
	Electrode material	Gold
	Sensing material	PDMS, polyurethane rubber, and silicone rubber
	Sensing layer dielectric constant	2.69, 3, and 3.7, respectively
	Electrode bulk conductivity (S/m)	2.5 × 10^−14^, 1 × 10^−11^, and 3.47 × 10^−4^, respectively
Square	Electrode length (a)	0.5 mm
	Electrode width (b)	0.5 mm
	Electrode thickness (S)	20 µm
	Sensing layer thickness (t)	20 µm
	Electrode material	Gold
	Sensing material	PDMS, polyurethane rubber, and silicone rubber
	Sensing layer dielectric constant	2.69, 3, and 3.7, respectively
	Electrode bulk conductivity (S/m)	2.5 × 10^−14^, 1 × 10^−11^, and 3.47 × 10^−4^, respectively

**Table 2:** Comparison of proposed results versus existing results.

Parameter	[27] (Square with 4 Straight Slots, Poly-Si)	Proposed Design (Circular with 2 Crescents, PDMS)	Level of Superiority in Proposed Design
Sensitivity	1.05 fF/mmHg	10.68 fF/mmHg	10.2× higher
Maximum Deflection	8.09 μm	1.35 × 10^−2^ mm	1.7× higher
Initial Capacitance	0.195 pF	0.892 pF	4.6× higher
Final Capacitance	0.363 pF	2.733 pF	7.5× higher
Capacitance change	0.168 pF	1.841 pF	11.0× higher

**Table 3:** Dielectric constants and bulk conductivity of the selected dielectric materials.

S. No.	Dielectric Material	Dielectric Constant	Bulk Conductivity (S/m)
1	PDMS	2.69	2.5 × 10^−14^
2	Polyurethane Rubber	3.0	1 × 10^−11^
3	Silicone Rubber	3.70	3.47 × 10^−4^

**Table 4:** Capacitance values (theoretical and simulated) for solid circular and square-shaped electrodes with PDMS sensing layer.

Sensor Type	Applied Pressure (mmHg)	Theoretical Capacitance Value (pF)	Simulated Capacitance Value (pF)	Mean Deviation in Capacitance, ΔC (pF)
Solid Circular-Shaped Electrode	0	0.93	0.95	0.02
	60	1.02	1.04	0.02
	200	1.30	1.32	0.02
Solid Square-Shaped Electrode	0	0.29	0.33	0.04
	60	0.32	0.36	0.04
	200	0.40	0.44	0.04

**Table 5:** Parametric values of the proposed sensor structures for selected dielectric materials.

Plate Type	Slot Type	Plate Material	Slot Inner Arc/Length (mm)	Slot Width (mm)	Deflection Maximum (mm)	Capacitance Without Deflection (pF)	Capacitance With Maximum Deflection (pF)
Circular	No slots	PDMS	--	--	5.648 × 10^−3^	0.95063	1.324
		Polyurethane Rubber	--	--	1.3000 × 10^−4^	1.0585	1.0656
		Silicone Rubber	--	--	4.6210 × 10^−4^	1.4055	1.4386
	Two Crescents	PDMS	0.7330	0.05	1.35 × 10^−2^	0.89248	2.7334
		Polyurethane Rubber	0.7330	0.05	4.56 × 10^−4^	0.98935	1.0127
		Silicone Rubber	0.7330	0.05	1.8219 × 10^−3^	0.36336	0.3987
	Four Crescents	PDMS	0.2749	0.05	1.5013 × 10^−2^	0.36423	1.4302
		Polyurethane Rubber	0.2749	0.05	4.76 × 10^−4^	1.0072	1.0314
		Silicone Rubber	0.2749	0.05	1.89 × 10^−3^	1.2321	1.3582
Square	No slots	PDMS	--	--	5.29 × 10^−3^	0.33203	0.45144
		Polyurethane Rubber	--	--	1.32 × 10^−4^	0.33203	0.33424
		Silicone Rubber	--	--	4.43 × 10^−4^	0.33203	0.33955
	Four I-Shaped	PDMS	0.25	0.0125	5.69 × 10^−3^	0.27902	0.39023
		Polyurethane Rubber	0.25	0.0125	1.58 × 10^−4^	0.3099	0.31236
		Silicone Rubber	0.25	0.0125	5.2796 × 10^−4^	0.40951	0.42061
	Four L-Shaped	PDMS	0.225	0.025	5.0606 × 10^−3^	0.11068	0.14817
		Polyurethane Rubber	0.225	0.025	1.2942 × 10^−4^	0.31211	0.31414
		Silicone Rubber	0.225	0.025	4.3597 × 10^−4^	0.38261	0.39114

**Table 6:** Sensitivity values of the designed sensors.

Sensor	Slots	Dielectric Material	Sensitivity (fF/mmHg)
Circular capacitive sensor	0	PDMS	2.01
		Polyurethane rubber	3.87857 × 10^−2^
		Silicone rubber	1.85429 × 10^−1^
	2	PDMS	10.68
		Polyurethane rubber	1.19443 × 10^−1^
		Silicone rubber	1.79943 × 10^−1^
	4	PDMS	6.23
		Polyurethane rubber	1.24429 × 10^−1^
		Silicone rubber	6.46071 × 10^−1^
Square capacitive sensor	0	PDMS	6.46925 × 10^−1^
		Polyurethane rubber	1.10536 × 10^−2^
		Silicone rubber	3.78679 × 10^−2^
	4	PDMS	6.12496 × 10^−1^
		Polyurethane rubber	1.235 × 10^−2^
		Silicone rubber	5.60036 × 10^−2^
	8	PDMS	2.02011 × 10^−1^
		Polyurethane rubber	1.01679 × 10^−2^
		Silicone rubber	4.29036 × 10^−2^

**Table 7:** Comparison of present work with previous studies.

Reference No.	Coil Shape	Inductance	Material of Sensor	Sensor Shape	Dimension of Sensor	Frequency	Sensitivity	Pressure Range
[24]	Stent Length: 7.2 mm	180 nH	Copper, silicon wafer, glycerol	Circular	Hydraulic chamber: 400 × 100 × 12 µm^3^	100 KHz–3 GHz	0.052 fF/mmHg	0–300 mmHg
[6]	Stent Length: 30 mm	350 nH	Gold, polyimide, silicon nitride	Ellipse	750 × 424 × 200 µm^3^	26.78–27.09 MHz	7.73 ff/mmHg	0–240 mmHg
[6]	Stent Length: 30 mm	350 nH	Gold, polyimide, silicon nitride	Circular	564 × 564 × 200 µm^3^	26.78–27.09 MHz	9.94 ff/mmHg	0–240 mmHg
[21]	Planar	1.2 µH	Glass, gold, silicon	Rectangular	2.6 × 1.6 mm^2^	95–103 MHz	120 KHz/mmHg	0–50 mmHg
[22]	Stent Length: 20 mm	530 nH	Stainless steel, parylene C	Square	1.5 × 1.5 × 0.2 mm^3^	50 MHz	146 ppm/mmHg	0–250 mmHg
Present Work	Stent Length: 30 mm	927 nH	Gold, PDMS	Circular	1000 × 1000 × 20 µm^3^	100 KHz–110 MHz	10.68 ff/mmHg	60–200 mmHg

## Data Availability

Data is contained within the article.
